# Long-Term Patient-Centered Outcomes After Congenital Syndactyly Reconstruction: Aesthetic, Functional, and Psychosocial Assessment

**DOI:** 10.3390/jcm15124815

**Published:** 2026-06-21

**Authors:** Zeynel Mert Asfuroğlu, Bengisu Özçivit Asfuroğlu, Elyesa Ergen, Emre Öztürk, Ender Gümüşoğlu, Metin Manouchehr Eskandari

**Affiliations:** 1 Division of Hand Surgery, Department of Orthopaedics and Traumatology, School of Medicine, University of Mersin, 33110 Mersin, Turkey; elyesa.ergen@gmail.com (E.E.); mavs137@gmail.com (E.Ö.); endergumusoglu@gmail.com (E.G.); mmeskandari@yahoo.com (M.M.E.); 2 Department of Child and Adolescent Psychiatry, Mersin City Training and Research Hospital, 33240 Mersin, Turkey; bengisuozcivit@gmail.com

**Keywords:** congenital hand anomalies, long-term outcomes, patient-centered outcomes, psychosocial impact, syndactyly, Withey score

## Abstract

**Background and Objectives:** Syndactyly is a common congenital hand anomaly that may affect hand appearance, function, and psychosocial well-being. This study aimed to evaluate long-term patient-centered outcomes after congenital syndactyly reconstruction, including aesthetic, functional, and psychosocial domains. **Methods:** This retrospective study included 53 patients with 90 reconstructed web spaces. Aesthetic outcomes were assessed using the Withey score, functional outcomes using the QuickDASH questionnaire, and psychosocial outcomes using an exploratory patient-centered survey developed by the authors. **Results:** The median follow-up duration was 10 years. The median outcome scores suggested generally favorable long-term results, with a Withey score of 2, a QuickDASH score of 14, and a psychosocial survey score of 29, all within the favorable range of their respective scales. Poorer aesthetic outcomes were observed in patients with complicated syndactyly, those who underwent surgery between 1 and 5 years of age, and those who underwent multiple surgeries. Female sex was associated with poorer functional and psychosocial scores. Complicated syndactyly was associated with less favorable outcomes across all domains. The psychosocial survey demonstrated high internal consistency and significant correlations with both functional and aesthetic outcomes. **Conclusions:** Congenital syndactyly reconstruction was associated with generally favorable long-term patient-centered outcomes. Less favorable results were observed particularly in patients with complicated syndactyly, while age- and surgery-related associations should be interpreted cautiously because of the retrospective design. These findings support the importance of individualized counseling and long-term assessment that includes aesthetic, functional, and psychosocial dimensions.

## 1. Introduction

Among congenital anomalies of the hand, syndactyly is one of the most common, with an estimated prevalence of 3 to 40 per 10,000 newborns [1,2]. This condition develops during the sixth week of gestation due to failure of apoptosis in the interdigital web spaces, a process that normally enables separation of the digits within the hand plate [3,4]. Syndactyly can be classified as complete (extending to the fingertip) or incomplete, and as simple (cutaneous) or complex (including bony fusion). It can present either as an isolated anomaly or in association with a syndrome [5]. Complicated syndactyly, involving accessory digits or abnormal bones within the fusion, is typically linked to conditions such as Apert and Poland syndromes [6,7].

Surgical release of syndactyly is performed to improve hand function and appearance and to prevent progressive deformity during growth. Children with congenital anomalies of the hand or upper limb, including syndactyly, may experience substantial psychosocial stress, as functional limitations may interfere with daily life and visible differences in appearance may influence social interactions and psychological well-being [8,9].

To date, the literature on syndactyly has focused predominantly on surgical reconstruction and functional outcomes [10,11,12]. By contrast, the psychosocial impact of upper-limb differences in the pediatric population has been less extensively studied [13,14,15]. Further insight into these effects may contribute to a more comprehensive evaluation of treatment outcomes and better inform postoperative counseling. Accordingly, long-term outcome assessment after syndactyly reconstruction should not be limited to technical or functional results, but should also include patient-centered domains such as appearance-related concerns and psychosocial well-being.

Various surgical techniques have been described for syndactyly release, including dorsal commissural flaps and zig-zag incisions, with or without the use of full-thickness skin grafting [6,7,10,11,12]. While these approaches generally aim to optimize both function and appearance, long-term outcomes that integrate aesthetic, functional, and psychosocial domains remain incompletely characterized. In particular, few studies have evaluated these domains together or examined which patient- and treatment-related factors may be associated with less favorable long-term results.

In this study, we evaluated the long-term patient-centered outcomes of congenital syndactyly reconstruction, including aesthetic, functional, and psychosocial domains. Psychosocial outcomes were assessed using an exploratory patient-centered survey, and patient- and treatment-related factors associated with these outcomes were also examined.

## 2. Materials and Methods

### 2.1. Patient Selection and Data Collection

**Figure 1:**
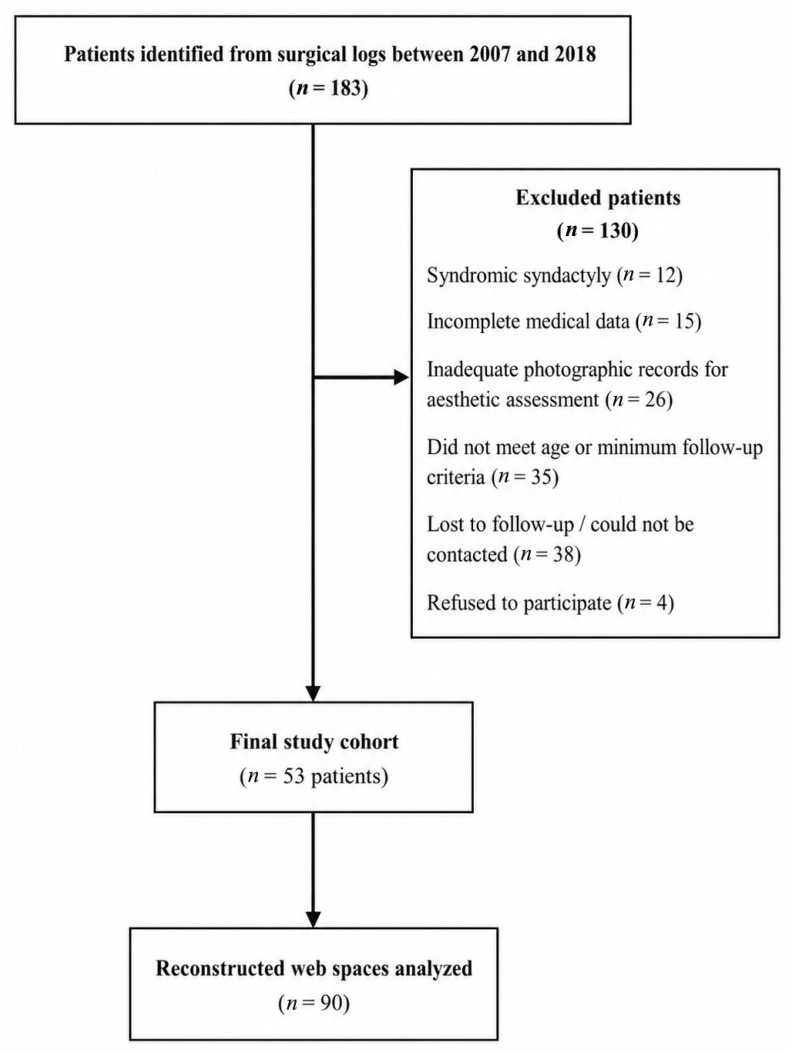
Patient selection flow diagram.

Before study initiation, the research protocol was approved by the institutional review board (approval no. 2024/875). Surgical logs from 2007 to 2018 at a single tertiary referral center were reviewed to identify patients diagnosed with syndactyly who underwent surgical reconstruction. In total, 183 patients were identified. After exclusions, loss to follow-up, and refusal to participate, the final study cohort consisted of 53 patients, and 90 reconstructed web spaces were analyzed. The patient selection process and reasons for non-inclusion are summarized in Figure 1. Patient assessment included a review of medical and photographic records. Written informed consent was obtained from all participants or their legal representatives. Additional informed consent for publication of clinical photographs was obtained from the patients and/or their legal representatives, as appropriate.

Patients were eligible for inclusion if they had undergone surgery before the age of 18 years, were at least 10 years old at the time of assessment, and had a minimum follow-up period of 5 years. Patients were excluded if they had incomplete medical data, inadequate photographic records for aesthetic assessment, syndromic syndactyly (e.g., Apert or Poland syndrome), or refusal to participate. Eligible patients were invited for a final follow-up assessment. At this visit, standardized photographs were obtained and the study questionnaires were administered. Ultimately, 90 web spaces from 53 patients were analyzed.

Medical records and demographic data—including age, sex, surgical details (age at surgery, anatomical location, surgical technique, number of surgical sessions, and postoperative follow-up time)—were collected through the hospital’s digital information system. Syndactyly release was performed using interdigitating zig-zag incisions for digital separation and an appropriately designed dorsal fasciocutaneous commissural flap for web-space reconstruction, such as rectangular, triangular, or omega-shaped dorsal commissural flaps according to the web-space anatomy. Full-thickness skin grafting was used when tension-free primary closure could not be achieved. In complex or complicated cases, additional procedures such as bony separation, excision of accessory tissue, or correction of associated deformity were performed as required. Multiple surgical sessions referred to more than one planned operative session, usually performed for multiple web-space involvement. Unplanned reoperations or revision procedures performed for complications, such as web creep or wound-related problems, were recorded separately and were not included in the multiple surgical session variable used for subgroup comparisons. Syndactyly classification was based on preoperative clinical and radiographic findings [5]. In patients with multiple forms of syndactyly, the most severe type was used for classification.

### 2.2. Postoperative Assessment Criteria

**Figure 2:**
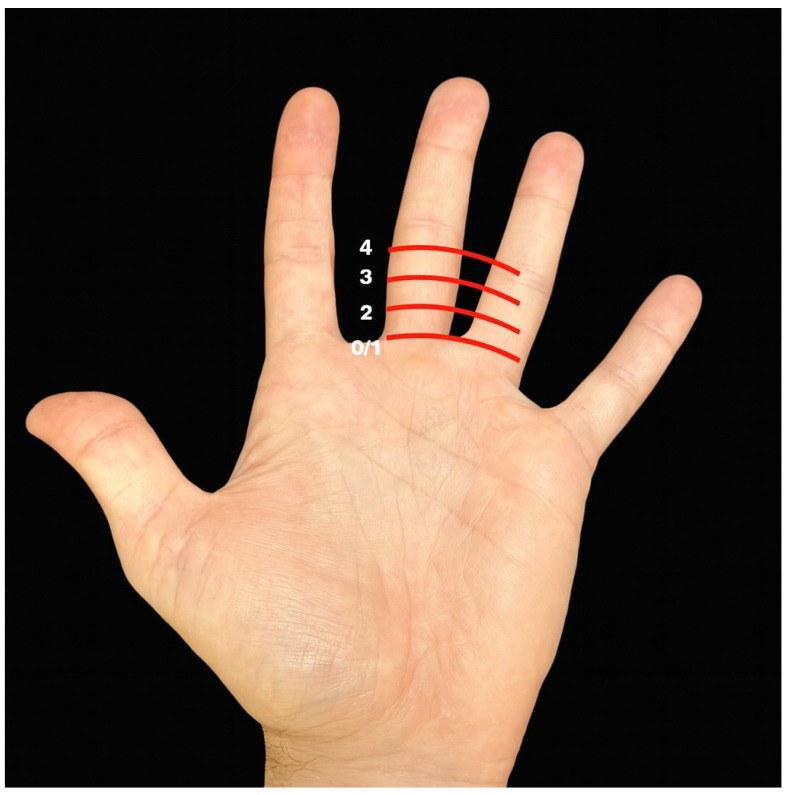
Evaluation of web creep using the Withey scoring system.

At the final follow-up visit, standardized photographs of both hands were obtained using a smartphone camera. To ensure consistency, photographs were taken under similar lighting conditions, from a similar distance, and against a uniform background. Palmar, dorsal, and lateral-view photographs were obtained for each patient. The appearance of the web space was assessed on these photographs using the Withey score [16] (Table 1, Figure 2). This scoring system ranges from 1 to 11, with 1 representing the best outcome and 11 the poorest. Scoring was performed independently by three observers (ZMA, EE, EG) on two separate occasions, with a one-week interval between assessments. The arithmetic mean of the observers’ scores was used to determine the final value. In patients with involvement of multiple web spaces, each web space was assessed individually, and the arithmetic mean of the web-space scores was recorded as the patient’s final Withey score.

Functional outcomes were evaluated using the Disabilities of the Arm, Shoulder and Hand (QuickDASH) questionnaire [17]. QuickDASH is a shortened version of the original DASH survey, consisting of 11 items rather than 30, and is used to assess physical function and symptoms in individuals with upper-limb disorders. The items assess upper-extremity activity limitations (e.g., opening a jar, carrying a shopping bag, washing one’s back), symptoms (pain, tingling), and the impact on daily activities/work. Scores range from 0 (best possible function) to 100 (poorest function).

For psychosocial assessment, an exploratory, author-developed patient-centered survey adapted for pediatric and adolescent participants was used (Table 2). The survey consisted of eight items scored from 1 to 4, yielding a total score range of 8 to 32, with lower scores indicating greater psychosocial impact and higher scores indicating less or no psychosocial impact. Both questionnaires were completed by the patients themselves; in younger participants, caregiver assistance was permitted only when necessary to facilitate comprehension.

### 2.3. Statistical Analysis

All statistical analyses were performed using SPSS software, version 26.0 (IBM Corp., Armonk, NY, USA). Categorical variables were presented as numbers and percentages [*n* (%)], whereas continuous variables were expressed as median and interquartile range [median (Q1–Q3)] because they did not show a normal distribution. The Mann–Whitney U test was used for comparisons of scores between two independent groups, while the Kruskal–Wallis test was used for comparisons among three or more independent groups. When a statistically significant difference was detected with the Kruskal–Wallis test, post hoc pairwise comparisons were performed using the Mann–Whitney U test, with Bonferroni correction applied for multiple comparisons. Adjusted analyses were performed using linear regression models. For each model, the relevant outcome score was entered as the dependent variable, and the variable of interest and syndactyly type were entered as independent variables. Adjusted β coefficients, 95% confidence intervals (CIs), and *p*-values were reported. These analyses were considered exploratory because of the small sample size and multiple subgroup comparisons. Inter-observer and intra-observer reliability of the Withey score were assessed using the intraclass correlation coefficient (ICC). ICC values were interpreted as follows: <0.40, poor; 0.40–0.59, fair; 0.60–0.74, good; and ≥0.75, excellent. Associations between psychosocial survey scores and both QuickDASH and Withey scores were evaluated using Spearman’s rank correlation coefficients. Internal consistency of the psychosocial survey was assessed using Cronbach’s alpha and McDonald’s omega coefficients. A *p*-value < 0.05 was considered statistically significant.

## 3. Results

### 3.1. Patient Demographic Characteristics

Of all patients, 28 (52.8%) were male. The median age at the time of surgery was 4 (2–8) years, the median age at the time of survey completion was 15 (12–18) years, and the median follow-up duration was 10 (8–14) years. Bilateral syndactyly reconstruction was performed in 16 (30.2%) patients. The most common type of syndactyly was simple–complete (41.5%), and the most frequently affected site was the third web space (55.5%). Multiple web-space involvement was observed in 31 (58.5%) patients, and 27 (50.9%) patients underwent multiple planned surgical sessions. Unplanned reoperation or revision for complications was required in 4 patients: 3 patients, all of whom had complicated syndactyly, underwent revision for web creep, and 1 patient required early reoperation for wound debridement. Full-thickness skin grafting was used for reconstruction in 29 (54.7%) patients. All demographic characteristics of the patients are presented in Table 3.

### 3.2. Results of Postoperative Assessment

**Figure 3:**
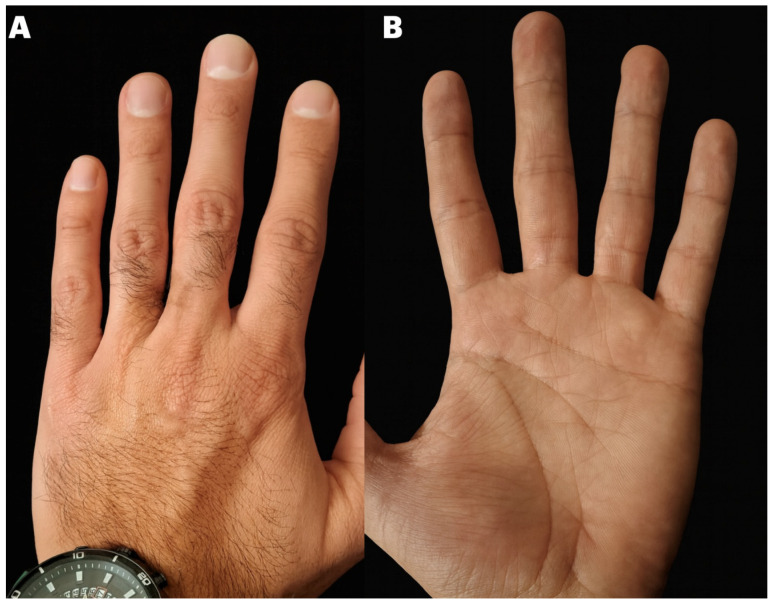
Clinical photographs of a patient with a favorable long-term outcome after third web-space complex syndactyly reconstruction at 16-year follow-up. Outcomes: Withey score = 1; QuickDASH = 11; psychosocial score = 31. (**A**) Dorsal view, (**B**) palmar view.

**Figure 4:**
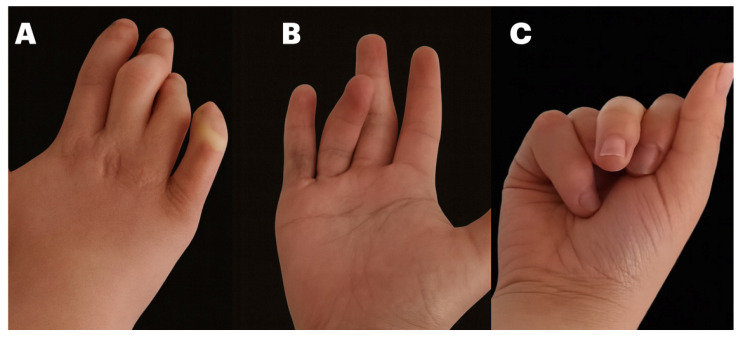
Clinical photographs of a patient with a less favorable long-term outcome after syndactyly reconstruction involving the second and third web spaces at 13-year follow-up; the patient had undergone multiple procedures for complicated syndactyly. Outcomes: Withey score = 8; QuickDASH = 26; psychosocial score = 21. (**A**) Dorsal view, (**B**) palmar view, (**C**) flexion view.

The median Withey score was 2 (1–5). The ICCs for interobserver reliability of the Withey score were 0.831 and 0.849 for each measurement, indicating excellent reliability. The ICCs for intra-observer reliability were 0.793, 0.811, and 0.765 for the three observers, also indicating excellent reliability. The median QuickDASH score was 14 (11–19). The median psychosocial survey score was 29 (24–31). Table 2 summarizes patient responses to the psychosocial assessment survey. Figure 3 and Figure 4 illustrate representative clinical photographs, showing a favorable and a less favorable long-term outcome.

Patients who underwent surgery between 1 and 5 years of age had poorer Withey scores than those who underwent surgery at other ages. Patients with complicated syndactyly had significantly poorer Withey scores than those with other syndactyly types. Patients who underwent multiple surgeries also had poorer Withey scores than those who underwent a single surgery (Table 4).

Female patients had significantly poorer QuickDASH scores than male patients. Patients with complicated syndactyly had significantly poorer QuickDASH scores than those with other syndactyly types (Table 4).

Female patients also had significantly poorer psychosocial survey scores than male patients. Additionally, patients with complicated syndactyly had significantly lower psychosocial survey scores than those with other types of syndactyly (Table 4).

Patient age at the time of survey completion, follow-up duration, bilateral involvement, multiple web-space involvement, and the use of skin grafting were not significantly associated with Withey, QuickDASH, or psychosocial survey scores (Table 4).

In adjusted linear regression analyses including syndactyly type, female sex remained associated with higher QuickDASH scores (β = 2.99, 95% CI: 0.24 to 5.74, *p* = 0.034) and lower psychosocial survey scores (β = −2.24, 95% CI: −4.36 to −0.12, *p* = 0.039). Surgery performed between 1 and <5 years of age remained associated with higher Withey scores compared with surgery performed between 5 and 8 years of age (β = 2.07, 95% CI: 0.72 to 3.42, *p* = 0.003), whereas surgery after 8 years of age was not significantly associated with Withey score (β = 0.22, 95% CI: −1.38 to 1.83, *p* = 0.779). Multiple surgeries also remained associated with higher Withey scores compared with a single surgery (β = 1.58, 95% CI: 0.44 to 2.72, *p* = 0.007).

### 3.3. Reliability of the Psychosocial Survey

McDonald’s omega and Cronbach’s alpha values for the psychosocial scale were 0.954 and 0.905, respectively, indicating high internal consistency. Psychosocial survey scores demonstrated a significant negative correlation with both QuickDASH scores (r = −0.862) and Withey scores (r = −0.616) (*p* < 0.05).

## 4. Discussion

In the present study, we evaluated the long-term patient-centered outcomes after congenital syndactyly reconstruction across aesthetic, functional, and psychosocial domains. Overall, the findings were generally favorable, with low disability scores, low psychosocial impact, and satisfactory aesthetic outcomes. Female sex was associated with poorer functional and psychosocial outcomes, whereas surgery performed between 1 and 5 years of age and a history of multiple surgeries were associated primarily with poorer aesthetic outcomes. The most consistent finding was that complicated syndactyly was associated with less favorable results across all outcome domains.

In our cohort, 52.8% of the patients were male and 47.2% were female. The predominant type of syndactyly was simple–complete, observed in 41.5% of patients. In addition, 30.2% of patients had bilateral involvement, and the third web space was the most commonly affected site (55.5%) (Table 3). These demographic characteristics are consistent with previous reports in the literature [1,2,3]. Although these findings are descriptive, they support the comparability of the present study population with previously reported syndactyly populations.

Withey et al. [16] developed a scoring system to assess outcomes after syndactyly release (Table 1). Kim et al. [18] reported that the Withey score has good inter-observer reliability and excellent intra-observer reliability when based on photographic assessment. In the present study, web space appearance was evaluated using the Withey score, which demonstrated excellent intra- and inter-observer reliability. Previous studies have shown that syndactyly severity and the need for revision surgery correlate with poorer aesthetic outcomes [19,20,21]. However, the literature presents mixed findings regarding the relationship between age at the time of surgery and postoperative finger appearance [20,21,22]. In our study, aesthetic outcomes were worse in patients with complicated syndactyly, those who underwent surgery between 1 and 5 years of age, and those who had multiple surgeries. The association between complicated syndactyly and poorer aesthetic outcome is clinically plausible, as greater anatomical complexity may increase the risk of residual deformity, scarring, web creep, or the need for revision procedures. The relationship between age at surgery and aesthetic outcome should be interpreted more cautiously. Surgical timing in retrospective series may reflect several factors, including referral patterns, case complexity, surgeon preference, and family-related factors, rather than an independent biological effect of age alone. Therefore, this finding should not be interpreted as evidence that surgery between 1 and 5 years of age is intrinsically associated with poorer outcomes, but rather as a potential marker of underlying clinical or treatment-related complexity. Remaining growth may also influence web-space remodeling and the risk of web creep, as school-aged children may retain greater soft-tissue adaptation potential than children older than 8 years; however, this mechanism was not directly assessed in the present retrospective cohort. Similarly, the association between multiple surgeries and poorer Withey scores should be interpreted cautiously, as multiple surgical sessions in this cohort mainly reflected multiple web-space involvement rather than revision surgery or reoperation for complications.

Several patient-reported outcome measures have been used to assess upper-extremity function after syndactyly reconstruction, among which the QuickDASH is one of the most widely used [17,20]. Although the QuickDASH is a general upper-extremity disability measure and is not specific to syndactyly, previous studies have supported its use in older children and adolescents with upper-extremity conditions [23,24]. In the present study, the median age at survey completion was 15 years, and all patients were at least 10 years old at the time of assessment. Therefore, the QuickDASH was considered suitable for providing a general assessment of upper-extremity disability in this cohort. Previous studies using patient-reported outcome measures have generally suggested that long-term functional impairment is limited and that overall quality of life is not markedly compromised after syndactyly reconstruction [20,25]. In the present study, the low median QuickDASH score similarly indicated that upper-extremity function and daily activities were generally well preserved in most patients. Nevertheless, poorer functional outcomes were associated with female sex and complicated syndactyly. The association with complicated syndactyly is not unexpected, as greater anatomical severity may lead to more residual stiffness, deformity, or dissatisfaction, which may in turn be reflected in patient-reported functional scores. The reason for the association between female sex and poorer QuickDASH scores remains uncertain. This finding should be considered exploratory, as our study was not designed to determine the mechanism underlying this sex-related difference. One possible explanation is that sex-related differences in symptom perception or self-reported upper-extremity disability may have influenced patient-reported assessments [26]; however, this interpretation should be made cautiously. In addition, because the QuickDASH is not syndactyly-specific, it may not fully capture subtle functional limitations related to web-space reconstruction, appearance-related hand use, or pediatric/adolescent-specific concerns.

The psychosocial impact refers to the effect of environmental and/or biological factors on an individual’s social and psychological characteristics [27]. Currently, there is limited information on the psychosocial profiles of individuals with syndactyly. In this study, psychosocial impact was assessed using an exploratory, author-developed patient-centered survey (Table 2), which was adapted from the same core survey previously used to evaluate the social impact of finger amputation [28]. Minor wording modifications were made to include school-related contexts for pediatric and adolescent participants. The survey focused on reactions to hand appearance in daily life, particularly in work or school settings, as well as the degree of personal discomfort. In the present cohort, the survey demonstrated high internal consistency and significant correlations with functional and aesthetic outcome measures. However, these findings should not be interpreted as evidence of formal validation, as internal consistency does not establish construct validity, criterion validity, or responsiveness. Therefore, the psychosocial findings should be regarded as exploratory, and further validation of this instrument in syndactyly-specific populations is required.

McDougall et al. [9] reported that psychosocial coping mechanisms may be effective in individuals with congenital hand and upper-limb differences. A literature review by Shah et al. [29] noted that patients with congenital upper-limb differences often have better peer relationships and increased adaptability. Bae et al. [30] suggested that children’s adaptability contributes to more positive psychosocial attitudes and behaviors. In our study, analysis of the psychosocial survey responses showed that most patients did not experience negative impacts on their daily life, work, or school following syndactyly reconstruction (Table 2). However, poorer psychosocial survey scores were associated with female sex and complicated syndactyly (Table 4). Although direct evidence specific to syndactyly is limited, previous studies on congenital hand and upper-limb differences have shown that visible differences in appearance may have important psychosocial consequences and may influence social interactions and self-perception [8]. In addition, females have been reported to experience greater body dissatisfaction than males [31]. Therefore, the lower psychosocial scores associated with female sex in our cohort may reflect differences in appearance perception or body image concerns; however, this explanation remains speculative and should be interpreted cautiously, as our study was not designed to determine the mechanism underlying this association. Taken together, these findings suggest that psychosocial assessment may add meaningful information to long-term follow-up after syndactyly reconstruction, particularly in patients with complicated deformities or greater appearance-related concerns.

There were several limitations to this study. First, the relatively small sample size and the absence of a concurrent control group limit the generalizability of our findings. In addition, because only patients who could be contacted and agreed to participate in the final follow-up assessment were included, selection bias cannot be excluded. Patients with particularly favorable or unfavorable outcomes may have been more or less likely to participate, which should be considered when interpreting the findings. Second, although web space distribution was recorded, outcomes were not analyzed according to specific web spaces due to the very small numbers in some subgroups, particularly the first web space. Third, the distribution of patients across some comparison groups was uneven, which may have influenced subgroup analyses. However, additional adjusted analyses were performed to examine whether the main associations persisted after accounting for syndactyly type distribution, and the key findings remained significant. Fourth, surgical technique-related differences were not analyzed, as this was beyond the scope of the study. Therefore, the present findings should not be interpreted as comparing the effectiveness of different operative techniques. Fifth, our survey targeted only patients who had undergone syndactyly reconstruction; parental perceptions and broader societal perspectives were not included. Sixth, although the QuickDASH has been used in older children and adolescents with upper-extremity conditions, it is a general upper-extremity disability measure and is not specific to syndactyly. Therefore, it may not fully capture subtle syndactyly-specific functional limitations, particularly in pediatric and adolescent patients. Seventh, psychosocial assessment was based on an author-developed survey that was not formally validated for patients with syndactyly. Although internal consistency was high, construct validity, criterion validity, and responsiveness were not assessed; therefore, the psychosocial findings should be interpreted cautiously and considered exploratory. Finally, we could not directly compare our findings with those of patients who were managed non-operatively or those who had not yet undergone surgery. Despite these limitations, the strengths of this study include its long-term follow-up period and the inclusion of psychosocial assessment.

## 5. Conclusions

In conclusion, this long-term follow-up study suggests that congenital syndactyly reconstruction is associated with generally favorable long-term patient-centered outcomes across aesthetic, functional, and psychosocial domains in this cohort. Less favorable results were observed particularly in patients with complicated syndactyly, which may reflect the greater anatomical and treatment-related complexity of this subgroup. The associations between surgery performed between 1 and 5 years of age, multiple surgeries, and poorer aesthetic outcomes should be interpreted cautiously, as these findings may reflect referral patterns, case complexity, surgeon preference, institutional logistics, or family-related factors rather than a direct effect of surgical timing or number of procedures. Female sex was associated with poorer functional and psychosocial scores; however, this exploratory association should be interpreted cautiously, as the study was not designed to determine the underlying mechanism. These findings highlight the importance of individualized treatment planning, careful preoperative counseling, and comprehensive postoperative support, including psychosocial counseling, especially for patients at higher risk of unfavorable outcomes. Given the retrospective design and relatively small sample size of this study, further prospective studies are needed to confirm these associations and to better define long-term patient-centered outcomes after syndactyly reconstruction.

## Figures and Tables

**Table 1:** Withey score for aesthetic assessment.

Parameter	Grade	Description
**Scar quality**	1	Thin and narrow
	2	Wide and flat
	3	Raised and thick
**Flexion–extension deformity**	0	Normal digit
	1	Finger cannot be hyperextended
	2	Finger has a fixed flexion deformity
**Web creep** **(** Figure 2 **)**	0	Soft web, abduction mirrors the adjacent web or equivalent web on the other hand
	1	No web advancement, but thickening of the web with reduced span
	2	Creep of web to 1/3 of the distance between base of the web and PIPJ crease
	3	Creep of web to 2/3 the distance between base of the web and PIPJ crease
	4	Creep of web to the PIPJ crease
**Lateral flexion deformity**	0	Absent
	1	Present
**Rotation deformity**	0	Absent
	1	Present

PIPJ = proximal interphalangeal joint.

**Table 2:** The survey developed for psychosocial assessment and the responses provided by the patients.

**1. Do you need to hide your hand during daily life? (for example; with gloves or long sleeves)**		
		*n* (%)
Yes, always	1	1 (1.9)
Yes, often	2	10 (18.9)
Seldom	3	7 (13.2)
No, never	4	35 (66)
**2. How often do your first-time contacts ask the question “what happened to your hand”?**		
		*n* (%)
At least once every day	1	6 (11.3)
Often, even if not every day	2	12 (22.6)
Seldom	3	25 (47.2)
I don’t remember being asked at all	4	10 (18.9)
**3. Do you use your affected hand in activities that may involve contact with other people’s hands, such as shaking hands or giving/receiving items?**		
		*n* (%)
I never use	1	2 (3.8)
I try not to use as much as possible	2	4 (7.5)
Sometimes I hesitate, but use it often	3	17 (32.1)
I always use without hesitation	4	30 (56.6)
**4. Do you feel that other people are disturbed by the appearance of your hand during daily activities that make your hand visible, such as shaking hands or giving/receiving items?**		
		*n* (%)
Yes, always	1	3 (5.7)
Yes, often	2	9 (17)
Seldom	3	12 (22.6)
No, never	4	29 (54.7)
**5. Are you currently studying or working in a job?**		
		*n* (%)
No, the cause is purely due to the appearance and/or dysfunction of my hand	1	0 (0)
No, the cause is mostly due to the appearance and/or dysfunction of my hand	2	0 (0)
No, but the cause is not about my hand	3	0 (0)
Yes	4	53 (100)
**6. How did your hand problem affect you in terms of your profession or school life?**		
		*n* (%)
I have serious problems because of the appearance of my hand rather than its dysfunction.	1	1 (1.9)
I have problems because of both the dysfunction and appearance of my hand	2	16 (30.2)
I have problems because of the dysfunction of my hand rather than its appearance	3	3 (5.7)
Neither the appearance nor the dysfunction of my hand is a problem	4	33 (62.3)
**7. Has there been a change in your job or school after syndactyly surgery?**		
		*n* (%)
Yes, the cause is purely due to the appearance and/or dysfunction of my hand	1	0 (0)
Yes, the cause is mostly due to the appearance and/or dysfunction of my hand	2	0 (0)
Yes, but the cause is not about my hand	3	2 (3.8)
No	4	51 (96.2)
**8. Do you feel that you receive different reactions from other people, such as customers, colleagues, supervisors, or teachers, because of the appearance of your hand at work or school?**		
		*n* (%)
Yes, always	1	4 (7.5)
Yes, often	2	10 (18.9)
Seldom	3	29 (54.7)
Never before	4	10 (18.9)

**Table 3:** Patient demographic characteristics.

**Gender, _n (%)_**	
*Male*	28 (52.8)
*Female*	25 (47.2)
**Age at the time of surgery, _median (Q1–Q3)_**	4 (2–8)
**Age at the time of survey completion, _median (Q1–Q3)_**	15 (12–18)
**Follow-up duration (year), _median (Q1–Q3)_**	10 (8–14)
**Side, *_n_* _(%)_**	
*Right*	15 (28.3)
*Left*	22 (41.5)
*Bilateral*	16 (30.2)
**Syndactyly Classification, *_n_* _(%)_**	
*Simple–Incomplete*	11 (20.8)
*Simple–Complete*	22 (41.5)
*Complex*	12 (22.6)
*Complicated*	8 (15.1)
**Location, *_n_* _(web-space, %)_**	
*1st web*	2 (2.2)
*2nd web*	23 (25.6)
*3rd web*	50 (55.5)
*4th web*	15 (16.7)
**Affected Web spaces, _*n* (patient, %)_**	
*Single*	22 (41.5)
*Multiple*	31 (58.5)
**Number of planned surgical sessions, *_n_* _(patient, %)_**	
*Single*	26 (49.1)
*Multiple*	27 (50.9)
**Need for skin grafting, *_n_* _(patient, %)_**	
*Yes*	29 (54.7)
*No*	24 (45.3)

**Table 4:** Results of postoperative assessment.

	QuickDASH [Median (Q1–Q3)]	Withey [Median (Q1–Q3)]	Psychosocial [Median (Q1–Q3)]
**Gender**			
Male (*n* = 28)	12 (11–16.5)	2 (1–4.25)	30 (27.75–31)
Female (*n* = 25)	18 (13–26)	3 (2–6)	26 (21–30)
*p*-value	**0.0036**	0.1648	**0.0211**
**Age at the time of surgery**			
1 to <5 years (*n* = 27)	18 (12–22.5)	4 (2–6) ^A^	27 (21–30)
5 to 8 years (*n* = 13)	13 (11–13)	2 (1–2)	31 (30–31)
>8 years (*n* = 13)	14 (11–23)	2 (1–3)	30 (23–30)
*p*-value	0.265	**0.008**	0.075
**Age at the time of survey completion**			
10–14 (*n* = 24)	13 (11–16.75)	2.5 (1–4.25)	30 (26.25–31)
≥15 (*n* = 29)	17 (12–23)	2 (2–5)	28 (23–30)
*p*-value	0.107	0.848	0.120
**Follow-up time (year)**			
<10 (*n* = 26)	13 (11–18.25)	2 (1–4)	30 (25–31)
≥10 (*n* = 27)	17 (12.5–21)	3 (2–6)	27 (23.5–31)
*p*-value	0.165	0.310	0.249
**Side**			
Unilateral (*n* = 37)	13 (11–19)	3 (1–4)	29 (24–31)
Bilateral (*n* = 16)	14 (11–18.75)	2 (1.75–6.5)	30 (24.75–31)
*p*-value	0.737	0.890	0.583
Syndactyly Classification *			
Simple–Incomplete (*n* = 11)	11 (11–12)	2 (1–2)	30 (28.5–31)
Simple–Complete (*n* = 22)	14 (11.25–17.75)	2 (1–4)	30 (26.75–30.75)
Complex (*n* = 12)	13 (12.5–18.75)	3 (2–4.25)	29 (24–31)
Complicated (*n* = 8)	26.5 (23.75–27.5) ^B^	6 (5.5–8) ^B^	20.5 (18.25–23) ^B^
*p*-value	**<0.001**	**0.002**	**0.002**
**Affected Web-space**			
Single (*n* = 22)	12.5 (11–18)	2 (1–3.75)	30 (25.25–31)
Multiple (*n* = 31)	16 (13–23.5)	3 (2–6)	29 (23–30)
*p*-value	0.090	0.073	0.329
**Number of planned surgical sessions**			
Single (*n* = 26)	13.5 (11–18.75)	2 (1–3)	30 (25.25–31)
Multiple (*n* = 27)	14 (11.5–25)	4 (2–6)	29 (21–30)
*p*-value	0.355	**0.010**	0.336
**Need for skin grafting**			
Yes (*n* = 29)	13 (11–18.25)	2 (1–6)	29 (24–31)
No (*n* = 24)	13 (11–18.25)	2.5 (2–4)	30 (23.25–31)
*p*-value	0.255	0.891	0.606
**TOTAL**	14 (11–19)	2 (1–5)	29 (24–31)

Q1–Q3: interquartile range (first to third quartile), *n*: number; ^A^: Indicates statistical significance compared with the 5 to 8 years group. ^B^: Indicates statistical significance compared with the simple–incomplete, simple–complete, and complex groups. *: Two patients had different types of syndactyly. The more severe type was documented as the patient’s classification.

## Data Availability

The data presented in this study are available on request from the corresponding author due to privacy and ethical restrictions.
